# Long-Term Use of Antibiotics and Risk of Parkinson's Disease in the Nurses' Health Study

**DOI:** 10.1155/2020/4038375

**Published:** 2020-01-20

**Authors:** Natalia Palacios, Éilis J O'Reilly, Michael A. Schwarzschild, Alberto Ascherio

**Affiliations:** ^1^University of Massachusetts, Lowell, MA, USA; ^2^Department of Nutrition, Harvard T. H. Chan School of Public Health, Boston, MA, USA; ^3^School of Public Health, University College Cork, Cork, Ireland; ^4^Department of Neurology, Harvard Medical School and Massachusetts General Hospital, Boston, MA, USA; ^5^Channing Division of Network Medicine, Department of Medicine, Brigham and Women's Hospital and Harvard Medical School, Boston, MA, USA

## Abstract

**Objective:**

Antibiotic use is one of the strongest environmental predictors of an altered and less diverse gut microbiome, which has been linked to Parkinson's disease. To our knowledge, no prior study has examined the association between long-term antibiotic use and Parkinson's disease.

**Design:**

We conducted a prospective study of 59,637 women in the Nurses' Health Study who reported total duration of antibiotic use at ages 20–39, 40–59, 60 +, or during the past 4 years. We used Cox Proportional Hazard regression to estimate hazard ratios and 95% confidence intervals for the association between categories of antibiotic use and risk of PD.

**Results:**

One hundred and eighty cases of PD were confirmed during the follow-up. Self-reported antibiotic use at ages 20–39, 40–59, and 60 +, as assessed in 2004, was not significantly associated with PD risk in our cohort. The hazard ratio comparing participants who used antibiotics for 2 or more months vs. 1–14 days at age 20–39 was 0.98 (95% CI: 0.54, 1.78), at age 40–59 was 1.44 (95% CI: 0.88, 2.33), and at age 60 +was 0.88 (95% CI: 0.53, 1.47). Antibiotic use during the past four years, as assessed in 2008, was also not associated with future risk of PD (HR: 1.14, 95% CI: 0.62, 2.10).

**Conclusion:**

In this cohort study, we did not observe a significant association between antibiotic use and incidence PD. A major limitation of our study is assessment of exposure, which required many participants to recall their antibiotic use decades in the past. Thus, although the results of this study do not support an effect of antibiotic use on PD risk, larger investigations relying on records of antibiotic prescriptions would provide more definitive evidence.

## 1. Introduction

The involvement of the gastrointestinal system in Parkinson's disease (PD) is increasingly appreciated, and PD is now recognized as a systemic disease. A number of studies have reported alterations in the gut microbiome in PD patients compared with controls. Alterations in specific genera as well as reductions in the overall bacterial diversity of the gut microbiome have been observed in PD [1].

Antibiotic use is associated with rapid changes in taxonomic richness, diversity, and evenness of the gut microbiome [2] and opens up the opportunity of invasion by pathogenic bacteria [3]. Furthermore, antibiotic administration has been shown to alter the metabolism, protein activity, and gene expression of the microbiota [4]. Although, in healthy individuals, the microbiota generally partially recovers after antibiotic treatment has stopped, antibiotic-induced changes can persist for years [2]. The changes in the gut microbiome induced by antibiotic administration have been shown to be similar to those seen in persons with chronic diseases, and gastrointestinal inflammation, which can be induced by antibiotics, has been observed in PD [5].

Long-term use of antibiotics, especially early in life, has been linked with obesity, allergies, and other chronic disorders [6–9]. In the Nurses' Health Study (NHS), self-reported retrospective history of antibiotic use has been linked to increased risk of colorectal adenoma [10]. In this study, we explored among participants in the NHS whether use of antibiotics contribute to predict risk of PD.

## 2. Methods

### 2.1. Study Population

The NHS cohort was established in 1976 when 121,700 female-registered nurses enrolled by filling out a mailed health-related baseline questionnaire. Since baseline, NHS participants have completed mailed follow-up questionnaires every two years, reporting their health habits and disease outcomes, including PD. Follow-up rates have averaged over 90% at each follow-up cycle in this cohort. All subjects provided informed consent to participate in the study and the research project was approved by the Institutional Review Board (IRB) at Harvard Medical School.

### 2.2. Exposure Ascertainment

On the 2004 follow-up questionnaire, NHS participants answered the following question: “for each of the following periods of your life, please add up the total amount of time you used antibiotics (excluding skin creams, mouthwash, and isoniazid)” for time periods between ages 20–39, 40–59, and 60 +. The response categories consisted of “none,” “less than 15 days,” “15 days to 2 months,” “2–4 months,” “4 months–2 years,” “2-3 years,” “3–5 years,” and “5 + years.” The participants also reported the most common reason for the antibiotic use, including respiratory infection, chronic bronchitis, urinary tract infection, dental, acne/rosacea, and other reasons.

On the 2008 questionnaire, participants were asked “during the past 4 years, what is the total amount of time you used antibiotics (excluding skin creams, mouthwash or isoniazid)?” They were also asked to report the most common reason for antibiotic use: respiratory infection, chronic bronchitis, UTI, dental, acne/rosacea, and other reasons.

### 2.3. Ascertainment of PD Cases

PD identification and diagnostic confirmation procedures in the NHS have been described in prior publications [11]. Briefly, we asked NHS participants to self-report incident PD on biennial questionnaires. When a study participant self-reported PD, we sought permission from the study participant to contact their treating neurologist and obtain a medical record. If such permission was granted, we asked the treating physician for confirmation of the diagnosis and a copy of the patient's medical record. A neurologist specialized in movement disorders (M.A.S.) reviewed the medical records and assessed whether a diagnosis of PD was definite, probable, possible, or not indicated. If a medical record could not be obtained, participants were considered to have PD if the treating neurologist or internist indicated that the diagnosis of PD is definite or probable. Only definite or probable cases were included in our analyses.

### 2.4. Statistical Analysis

In the first set of analyses, we examined, using Cox proportional hazards regression, the association between self-reported antibiotic use at ages 20–39, 40–59, and 60 +, reported in 2004, and risk of incident PD between 2004 and 2012. We calculated the age and multivariate-adjusted hazard ratios (HR) with 95% confidence intervals (95% CI) of PD according to category of antibiotic use with low antibiotic use (1–14 days) as the reference category because this category contained the greatest number of PD cases. Person-time based on age in months was accumulated from baseline (2004) until the date of first PD symptoms, death, last completed questionnaire, or end of follow-up (June 2012), whichever came first. Multivariate analyses were adjusted for age in months, smoking (never or ever), pack-years smoking and postmenopausal hormone (PMH) use (never, past, or current) at baseline in 2004.

In subsequent analyses, we examined the association between antibiotic use over the past four years, as reported in 2008 and subsequent risks of incident PD between 2008 and 2012. Due to a limited number of cases in these analyses, we calculated the age and multivariate-adjusted HR of PD comparing participants who reported any antibiotic use to those who reported no antibiotic use during the past four years. Person-time based on age in months was accumulated from baseline (2008) until the date of first PD symptoms, death, last completed questionnaire, or end of follow-up (June 2012), whichever came first. Multivariate analyses were adjusted for age in months, smoking (never or ever) pack-years and postmenopausal hormone (PMH) use (never, past, or current) at baseline.

## 3. Results

The baseline characteristics of the study participants according to categories of antibiotic use are shown in Table 1. Participants reporting higher use of antibiotics at ages 20–39 and 40–59 were somewhat younger, while the opposite was true for those reported higher use of antibiotic at ages above 60. At all three age ranges surveyed, as well as for past four years of antibiotic use, a higher proportion of participants reporting 2 months or more of antibiotic use were current PMH users and a lower proportion of participants were non- PMH users, compared with those reporting lower levels of antibiotic use.

Overall, antibiotic use at ages 20–39, 40–59, and 60 + present was not related to risk of incident PD. The HR comparing participants who used antibiotics for 2 months or more with those who used them for only 1–14 days was 0.98 (95% CI: 0.54, 1.78) for the period of 20–39 years, 1.44 (95% CI: 0.88, 2.33) for 40–59 years, and 0.88 (95% CI: 0.53, 1.47) for 65 + years. We also did not observe significant associations between recent 4-year antibiotic use in 2008 and risk of incidence of PD between 2008 and 2012. The HR comparing participants who ever used antibiotics during the past four years, as reported in 2008, compared with participants who never used them was 1.14 (0.62, 2.10). The results of these analyses are presented in Table 2.

## 4. Discussion

We did not observe a significant association between retrospective self-reported use of antibiotics at any age between 20 and 60 +, as assessed in 2004, and risk of PD in participants of the Nurses' Health Study. We also did not observe a significant association between recent antibiotic use (during the past four years), as reported in 2008, and risk of PD between 2008 and 2012.

An important limitation of our study was assessment of exposure, which required many participants to recall their antibiotic use decades in the past. Further, we have limited information on the type and dose of antibiotics. And, the number of women who reported prolonged use of antibiotics, which is more likely to affect PD risk, was small, as reflected in the wide confidence intervals of our HR estimate. Thus, although the results of this study do not support an effect of antibiotic use on PD risk, larger investigations relying on records of antibiotics prescriptions would provide more definitive evidence.

## Figures and Tables

**Table 1:** Age-standardized characteristics of the Nurses' Health Study participants at baseline^a, b^ by the extent of antibiotic use.

	None	1–14 days	15 days to 2 months	More than 2 months	Missing
*Antibiotic use at age 20–39* ^*a*^					
Number of women	7,861	29,180	10,683	4,177	7,736
Age, years^b^	72.8 ± 6.8	69.3 ± 6.6	67.6 ± 6.5	66.6 ± 6.2	73.3 ± 6.6
Caffeine, mg/day	137.1 ± 125.4	139.6 ± 128.8	138.9 ± 127.4	134.8 ± 128.8	131.5 ± 127.0
Alcohol, g/day	5.8 ± 10.3	6.1 ± 10.8	6.2 ± 10.8	5.7 ± 10.9	5.5 ± 10.0
Pack-years smoking	11.8 ± 19.1	13.1 ± 19.9	12.3 ± 19.0	12.5 ± 19.4	13.0 ± 20.2
Nonsmoker, %	48.4	44.3	44.5	44.0	46.0
Past smoker, %	44.5	47.4	49.4	50.3	45.8
Current smoker, %	6.9	8.2	5.9	5.5	8.0
Non-PMH user, %	26.4	21.8	17.9	16.3	21.9
Past PMH user, %	52.6	55.8	57.8	56.6	55.4
Current PMH user, %	13.8	17.3	20.8	24.2	14.9

*Antibiotic use at age 40–59* ^*a*^					
Number of women	4,218	29,742	14,316	5,443	5,918
Age, years^b^	71.5 ± 6.9	69.8 ± 6.8	68.4 ± 6.8	67.5 ± 6.7	74.0 ± 6.2
Caffeine, mg/day	144.2 ± 129.4	140.5 ± 129.3	136.9 ± 125.9	129.3 ± 126.2	131.6 ± 128.5
Alcohol, g/day	6.2 ± 11.0	6.2 ± 10.9	5.8 ± 10.3	5.5 ± 10.3	5.3 ± 10.2
Pack-years smoking	11.8 ± 19.3	12.8 ± 19.5	12.7 ± 19.6	12.8 ± 19.7	13.0 ± 20.4
Nonsmoker, %	49.6	44.7	44.6	43.8	46.5
Past smoker, %	41.8	47.3	49.0	50.5	44.8
Current smoker, %	8.4	7.8	6.3	5.4	8.4
Non-PMH user, %	32.1	22.4	17.9	14.8	23.1
Past PMH user, %	48.3	55.6	58.1	57.7	53.7
Current PMH user, %	11.9	16.7	20.1	23.9	14.8

*Antibiotic use at age 60* *+*^*a*^					
Number of women	9,001	25,953	12,900	5,568	6,215
Age, years^b^	67.5 ± 6.6	70.8 ± 6.3	71.5 ± 6.0	72.5 ± 6.0	62.5 ± 6.8
Caffeine, mg/day	148.0 ± 133.3	140.0 ± 130.1	135.9 ± 125.1	128.7 ± 126.1	132.5 ± 123.8
Alcohol, g/day	6.4 ± 11.2	6.1 ± 10.6	5.5 ± 10.3	5.3 ± 9.9	5.3 ± 10.0
Pack-years smoking	12.2 ± 19.3	12.8 ± 19.5	13.3 ± 20.6	13.4 ± 20.3	11.9 ± 19.5
Nonsmoker, %	47.6	44.6	43.5	44.7	49.0
Past smoker, %	43.4	47.5	49.3	49.0	42.8
Current smoker, %	8.9	7.7	7.0	6.1	7.9
Non-PMH user, %	27.1	21.4	18.4	16.6	22.7
Past PMH user, %	52.7	55.9	57.6	56.0	54.8
Current PMH user, %	14.1	17.0	19.7	22.6	15.8

*Antibiotic use during the past four years* ^*b*^					
Number of women	13,467	22,343	13,470	4,163	330
Age, years^b^	73.9 ± 6.9	73.0 ± 6.7	72.6 ± 6.7	72.9 ± 6.9	78.6 ± 6.1
Caffeine, mg/day	11.0 ± 18.3	11.8 ± 18.8	12.9 ± 19.6	13.4 ± 20.3	12.2 ± 18.9
Alcohol, g/day	268.5 ± 220.5	269.2 ± 221.7	265.6 ± 224.7	259.3 ± 219.8	233.0 ± 209.0
Pack-years smoking	5.1 ± 9.2	4.9 ± 8.9	4.9 ± 8.8	4.7 ± 8.7	4.7 ± 8.7
Nonsmoker, %	45.1	48.1	51.0	51.8	47.4
Past smoker, %	6.0	5.7	5.2	4.5	6.4
Current smoker, %	48.7	46.1	43.7	43.5	46.2
Non-PMH user, %	25.6	20.5	16.9	14.6	21.3
Past PMH user, %	58.6	62.6	64.6	64.5	55.5
Current PMH user, %	9.2	11.6	14.0	16.1	10.2

Values in table are means (SD) or percentages and are standardized to the age distribution of the study population. ^a^Baseline is in 2004 for analyses of self-reported antibiotic use at ages 20–39, 40–59, and 60 + and in 2008 for analyses of past four years of antibiotic use. ^b^Value is not age-adjusted.

**Table 2:** Antibiotic use by duration of use at ages 20–39, 40–59, and 60 + in 2004 and part four years in 2008 in relation to subsequent PD risk in Nurses' Health Study.

	None	1–14 days	15 days to 2 months	More than 2 months	*p* trend

*Antibiotic use at age 20–39* ^*a*^					
Number of women	7,861	29,180	10,683	4,117	
PY	57,781	219,757	81,379	31,611	
Cases (*n* = 158)	19	104	23	12	
HR_crude_^c^ (95% CI)	0.68 (0.42, 1.10)	Ref	0.68 (0.43, 1.06)	1.01 (0.55, 1.83)	
HR_multiv_^d^ (95% CI)	0.67 (0.41, 1.09)	Ref	0.67 (0.43, 1.04)	0.98 (0.54, 1.78)	0.64
*Antibiotic use at age 40–59* ^*a*^					
Number of women	4,218	29,742	14,316	5,443	
PY	31,168	223,881	108,013	40,888	
Cases (*n* = 168)	12	91	45	28	
HR_crude_^c^ (95% CI)	1.01 (0.55, 1.84)	Ref	1.16 (0.82, 1.65)	1.45 (0.89, 2.35)	
HR_multiv_^d^ (95% CI)	1.00 (0.55, 1.83)	Ref	1.15 (0.81, 1.64)	1.44 (0.88, 2.33)	0.59
*Antibiotic use at age 60 +* ^*a*^					
Number of women	9,001	25,953	12,900	5,568	
PY	68,235	194,559	95,888	40,103	
Cases (*n* = 170)	19	93	40	18	
HR_crude_^c^ (95% CI)	0.70 (0.43, 1.15)	Ref	0.82 (0.57, 1.18)	0.89 (0.53, 1.47)	
HR_multiv_^d^ (95% CI)	0.70 (0.42, 1.15)	Ref	0.81 (0.56, 1.18)	0.88 (0.53, 1.47)	0.75

*Antibiotic use during the past four years (reported in 2008)* ^*b*^					
	Never	Ever	*p*		

Number of women	13,468	39,975			
PY	49,530	146,886			
Cases (*n* = 58)	14	44			
HR^crude^ (95% CI)^d^	Ref	1.14 (0.62, 2.09)			
HR^multiv^ (95% CI)^b^	Ref	1.14 (0.62, 2.10)	0.67		

^a^Reported in 2004; follow-up: 2004–2012. ^b^Reported in 2008; follow-up: 2008–2012. ^c^Adjusted for age as a continuous variable. ^d^Adjusted for age as a continuous variable, pack-years smoking (as a continuous variable), smoking (current, past, or never), and PMH use.

## Data Availability

The data used to support the findings of this study are restricted by the Partner's Healthcare Institutional Review Board in order to protect participant privacy. Data are available from the Nurses' Health Study for researchers who meet the criteria for access to confidential data.
